# Objective structured assessment of medical students’ technical skills in second-degree perineal laceration repair with sponge model-based training

**DOI:** 10.1007/s00404-023-07297-x

**Published:** 2023-12-11

**Authors:** Gregor Leonhard Olmes, Merle Doerk, Erich-Franz Solomayer, Meletios P. Nigdelis, Romina-Marina Sima, Bashar Haj Hamoud

**Affiliations:** 1 Department of Gynecology, Obstetrics and Reproductive Medicine, Saarland University Hospital, Kirrberger Straße 100, Building 9, 66421 Homburg, Saarland, Germany; 2 Carol Davila University of Medicine and Pharmacy, Bucharest, Romania

**Keywords:** Perineal laceration, OSATS, Medical teaching, Obstetrics, Student

## Abstract

**Purpose:**

In this cohort study, we used a sponge simulator to train students in second-degree perineal laceration repair. We examined whether the training course improved the students’ skills, as measured with an objective structured assessment of technical skills (OSATS) and by a senior physician. We also examined the correlation between these ratings to assess the validity of OSATS application in this context.

**Methods:**

Between April and July 2022, 40 medical students took part in gynecological/obstetrics training that included a lecture about perineal trauma and the viewing of a video that demonstrated second-degree perineal laceration repair using a sponge model. They then underwent initial evaluation by a senior physician and OSATS application, yielding two independent scores. After training with the sponge model, a second evaluation was performed. The OSATS assessed practical skills (8 items) and suture results (2 items). The senior physician assigned ratings on a five-point ordinal scale ranging from 1 (excellent) to 5 (poor).

**Results:**

Training with the sponge simulator significantly increased students’ OSATS (practical skills, *p* < 0.001; suture results, *p* < 0.05) and senior physician (*p* < 0.001) ratings. The OSATS and senior physician ratings correlated strongly (Spearman’s *r*: first assessment, – 0.72; second assessment, – 0.74; *p* < 0.01).

**Conclusion:**

The sponge-based training improves students’ skills for the repair of a second-degree perineal laceration. The OSATS for the sponge model might be a valid option to examine medical students in an obstetrical course.

## What does this study add to the clinical work

**Table:** 

This study provides insight on a structured training and examination approach for perineal laceration repair. Objective structured assessment of technical skills ratings correlated strongly with those of a senior physician	

## Introduction

Perineal laceration is a common obstetric injury, occurring in nearly 85% of women who deliver spontaneously [1]. Given this high prevalence, the establishment of an organized teaching model for medical and midwifery students is of great importance [2, 3]. The objective structured assessment of technical skills (OSATS) is an appropriate, widely approved method for medical skills assessment in gynecology [3, 4]. Previous studies of OSATS implementation for the assessment of perineal laceration repair skills have notable limitations, such as small numbers of participants and a focus on third- and fourth-degree lacerations [5–7]. We used a sponge model for perineal laceration repair training in an obstetrics and gynecology course. We examined whether this training module improved the students’ repair skills, as measured by OSATS application and by a senior physician. We assessed the validity of the OSATS as an evaluation tool relative to the senior physician’s assessment.

## Materials and methods

### Setting, participants, and procedure

Forty undergraduate students taking the obstetrics course provided by the Department of Gynecology, Obstetrics and Reproductive Medicine, Saarland University Hospital, Homburg (Saarland), Germany, between April and July 2022 were included in this study. A training module for the surgical treatment of second-degree perineal lacerations was implemented using a sponge model, along with evaluations by a senior physician and in form of OSATS (~ 10 min). The module began with a 10-min lecture about perineal lacerations and the technique used for their repair. The students then watched a video in which the continuous suture technique for second-degree perineal laceration repair was demonstrated step by step using the sponge model. They then underwent the first evaluation by OSATS administration and the senior physician. Thereafter, the students participated in a 30-min skills training session with the sponge simulator, followed by a second evaluation identical to the first. We included data from students who underwent all training and both evaluations in the final analysis; incomplete datasets were excluded.

### Sponge model

**Fig. 1:**
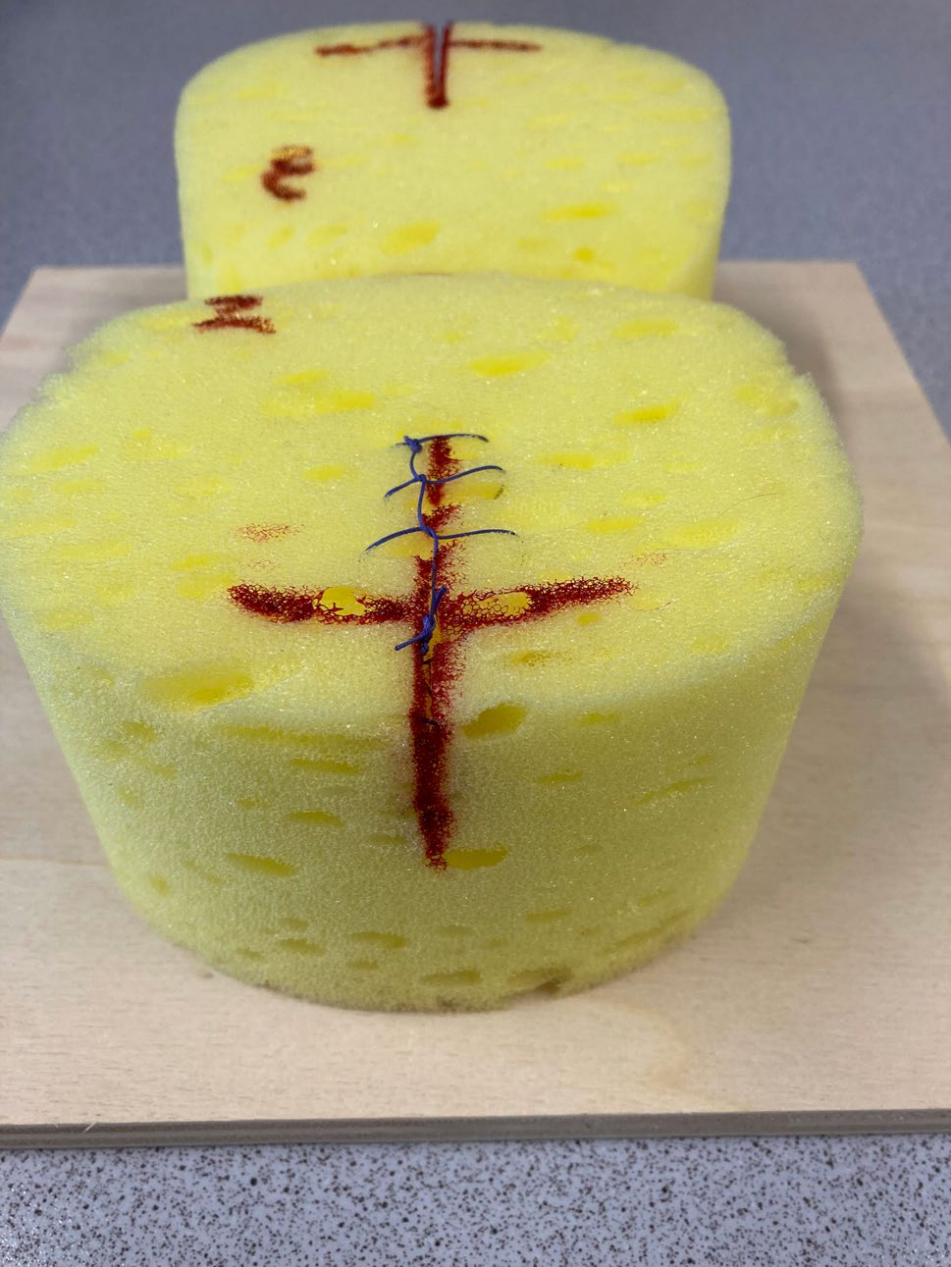
Sponge model trainer with performed suture, adapted from La Porte [8,9]

Conventional everyday material was used for the creation of the sponge model in an adaption of the technique described by La Porte  [8, 9]. For this model, a carwash sponge was fixed on a wooden board. At one end of the sponge, the foam was cut over a length of 5 cm (Fig. 1). A vertical red line was drawn to represent the vaginal and perineal laceration. Two red points were marked in the depth of the cut to represent the perineal muscle layer, and a transverse red line was drawn to represent the vaginal hymen [8, 9].

### OSATS

The OSATS consisted of two parts, the evaluation of the students’ practical skills and final suture results. Eight aspects of the students’ practical skills were evaluated: (1) the correct application of local anesthesia, (2) the initiation of the vaginal mucosa suture, (3) the creation of the first knot at the cranial end of the laceration, (4) the repair of the vaginal mucosa using a continuous locking suture, (5) termination at the vaginal hymen, (6) the suturing of the perineal body, (7) intracutaneous suturing, and (8) the creation of the finishing knot behind the vaginal hymen. Two aspects of the suture results were evaluated: stability (inability to insert forceps) and knot spacing (regular, 5–10 mm). One point was awarded for the correct performance of each aspect. The senior physician’s overall assessments were expressed on an ordinal scale ranging from 1 (excellent) to 5 (poor; Table 1).

**Table 1:** Evaluation of second-degree perineal laceration repair

OSATS
*Practical skills*
1. Correct application of local anesthetics
2. Initiation of vaginal mucosa suture
3. First knot at cranial end of laceration
4. Continuous locking suture of vaginal mucosa
5. Termination at vaginal hymen
6. Perineal body suturing
7. Intracutaneous suturing
8. Finishing with knot behind vaginal hymen
*Suture results*
1. Stability (inability to insert forceps)
2. Knot spacing (regular, 5–10 mm)
*Senior physician’s assessment*
1. Excellent
2. Good
3. Sufficient
4. Moderate
5. Poor

*OSATS* objective structural assessment of technical skills

## Data analysis

The explorative data analysis was performed by frequency tables. For analyzing the dataset of the evaluation by the OSATS and the senior physician Wilcoxon test was used. Spearman’s correlation coefficients were calculated to compare the OSATS and senior physician’s ratings.

Data were analyzed using SPSS software (version 25; IBM Corporation, Armonk, NY, USA). Statistical significance was defined as *p* < 0.05.

## Results

Data from 40 participating students were analyzed. The median practical skills’ rating on the first OSATS was 6. No student received 0–2 points and the largest number of students [*n* = 10 (25%)] received 7 points (Table 2). The median practical skills rating on the second OSATS was 8. No student received 0–3 points and the largest number of students [*n* = 26 (65%)] received 8 points (Table 2). The difference between the first and second median practical skills ratings was thus 2 points (*p* < 0.001).

**Table 2:** OSATS results (*n* = 40)

Practical skills	First assessment	Second assessment
0	0 (0%)	0 (0%)
1	0 (0%)	0 (0%)
2	0 (0%)	0 (0%)
3	7 (17.5%)	0 (0%)
4	4 (10%)	1 (2.5%)
5	8 (20%)	1 (2.5%)
6	5 (12.5%)	3 (7.5%)
7	10 (25%)	9 (22.5%)
8	6 (15%)	26 (65%)
Median (*p* < 0.001)	6	8
**Suture results**		
0	5 (12.5%)	1 (2.5%)
1	17 (42.5%)	10 (25%)
2	18 (45%)	29 (72.5%)
Median (*p* < 0.05)	1	2

*OSATS* objective structural assessment of technical skills

The median suture result rating on the first OSATS was 1. Five (12.5%) participants received no point and similar numbers of students received 1 [*n* =17 (42.5%)] and 2 [*n* =18 (45%)] points (Table 2). The median suture result rating on the second OSAT was 2. One (2.5%) student received no point and the largest proportion of students [*n* = 29 (72.5%)] received 2 points. The difference between the first and second median suture ratings was thus 1 point (*p* < 0.05).

The median rating from the senior physician’s first assessment was 3 (sufficient). Most ratings ranged from 2 (good) to 5 (poor), and four (10%) students received ratings of 1 (excellent; Table 3). The median rating from the senior physician’s second assessment was 1.5. No student received a rating of 5 (poor) and 20 (50%) students received a rating of 1 (excellent; Table 3). The difference between the first and second median ratings was 1.5 points (*p* < 0.001; Table 3).

**Table 3:** Senior physician’s ratings (*n* = 40)

Rating	First assessment	Second assessment
1 (excellent)	4 (10%)	20 (50%)
2 (good)	8 (20%)	7 (17.5%)
3 (sufficient)	9 (22.5%)	8 (20%)
4 (moderate)	9 (22.5%)	5 (12.5%)
5 (poor	10 (25%)	0 (0%)
Median (*p* < 0.001)	3	1.5

Spearman’s *r* values for the correlation between the OSATS and senior physician’s ratings were –0.71 for the first assessment and –0.74 for the second assessment. Both of these correlations were significant (*p* < 0.01).

## Discussion

In this study, training in perineal laceration repair using the sponge model improved the students’ skills, as measured by the OSATS and the senior physician. These two assessments correlated strongly, suggesting that OSATS application for this purpose is valid. OSATS use for the examination of medical students’ skills and course components in the field of gynecology and obstetrics has been validated [4]. Various models for the teaching of perineal repair, employing anatomical silicone, beef tongue, and sponges, have been established [10–12]. A cohort of residents provided positive feedback on a multimedia course in anal injury repair that included a video, slide shows, and training stations [13]. Other such courses in anal sphincter repair and episiotomy increased residents’ and midwifery students’ confidence and competence, respectively, in these techniques [11, 13].

Our results are line with data by Shah et al. [14] reporting on a similar laceration repair workshop. Their workshop significantly improved medical students’ knowledge (assessed by quizzes) and technical skills (knot-tying speed). Our training also significantly improved students’ practical skills, which we assessed in much greater detail.

An important aspect which needs to be taken into consideration regarding education research on perineal lacerations is that most studies have been conducted on residents [5, 6, 15, 16]. To some extent, extrapolation in the group of medical students is inevitable. In a randomized study, Dancz et al. [6] found that the respective use of beef tongue and sponge models significantly improved obstetrics residents’ confidence in and knowledge of the repair of third- and fourth-degree perineal lacerations, as determined by a 14-item task-specific checklist and global rating of general surgical skills, with no difference between models but residents’ preference for the beef tongue model. Their results for the sponge model are in line with our findings, although our sample was larger. Martinez et al. [5] found that a fourth-degree perineal laceration repair training course significantly improved 17 residents’ knowledge and performance of this procedure relative to that of 11 controls, as determined by a written test and an OSATS immediately after the course and repeated 6 months after. Their sample was smaller than ours, but they demonstrated a long-term effect of skills training, which we did not examine. In another randomized study, Banks et al. [15] determined that traditional teaching and a skills laboratory, respectively, significantly improved 24 medical residents’ knowledge and skills in second-degree perineal laceration repair, as determined by blinded physicians’ assessment with a task-specific checklist, a global rating scale, and pass/fail grade assignment. As in this study, they examined second-degree laceration repair skills, but they used a randomized trial, rather than cohort study, design.

Siddiqui et al. validated an OSATS for repair a of fourth-degree perineal lacerations simulated with a beef tongue model [16]. The validation was performed by three blinded judges, a task-specific OSATS, and a global rating scale [16]. Similarly, Siddighi et al. [7] demonstrated the construct validity of an OSATS with global surgical skills, procedure checklist, and global rating components for the evaluation of residents’ fourth-degree perineal laceration repair; 26 residents were included at baseline and 14 residents were reexamined, and showed improvement, 5 weeks after taking a workshop. Our study revealed a high correlation between the OSATS and the senior physician’s evaluation, but the model of fourth-degree laceration repair, use of blinded judges, and different checklists are some points of difference.

This study has limitations attributable to its design. The OSATS examined in the previous studies are not standardized for the second-degree perineal laceration repair; most are designed for the assessment of fourth-degree laceration repair [6, 7, 16], limiting the comparability of current and previous findings. In addition, we examined only the short-term effects of training, whereas 6-month effects of a workshop similar to our training course were also found to be positive [5].

## Conclusion

This study showed that training in the second-degree perineal laceration repair using a sponge model significantly improved students’ surgical skills. OSATS application was valid for the assessment of these skills in an obstetrical course, as reflected by the strong correlation of ratings with those of a senior physician.

## Data Availability

The dataset used and analyzed during the current study is available from the corresponding author on reasonable request.

## References

[1] O’Kelly SM, Moore ZE (2017). Antenatal maternal education for improving postnatal perineal healing for women who have birthed in a hospital setting. Cochrane Database Syst Rev.

[2] Diaz M, Simpson N, Brown A (2021). Effectiveness of structured education and training in perineal wound assessment and repair for midwives and midwifery students: a review of the literature. Eur J Midwifery.

[3] Eston M, Stephenson-Famy A, McKenna H, Fialkow M (2020). Perineal laceration and episiotomy repair using a beef tongue model. MedEdPORTAL.

[4] Hassan B, Elfaki O, Khan M (2017). The impact of outpatient clinical teaching on students’ academic performance in obstetrics and gynecology. J Fam Community Med.

[5] Martinez A, Cassling C, Keller J (2015). Objective structured assessment of technical skills to teach and study retention of fourth-degree laceration repair skills. J Grad Med Educ.

[6] Dancz CE, Sun V, Moon HB (2014). Comparison of 2 simulation models for teaching obstetric anal sphincter repair. Simulation in Healthcare.

[7] Siddighi S, Kleeman SD, Baggish MS (2007). Effects of an educational workshop on performance of fourth-degree perineal laceration repair. Obstet Gynecol.

[8] La Porte V (10.04.2012) Perineal repair simulation: materials and preparation. https://www.youtube.com/watch?v=yVnlP4WaTYE. Accessed 25 Aug 2022

[9] La Porte V (10.04.2012) 2nd degree perineal repair demonstration. https://www.youtube.com/watch?v=R4o4KSY4MMY. Accessed 25 Aug 2022

[10] Goudie C, Shanahan J, Gill A (2018). Investigating the efficacy of anatomical silicone models developed from a 3D printed mold for perineal repair suturing simulation. Cureus.

[11] Guler H, Cetin P, Yurtsal ZB (2018). Effect of episiotomy training with beef tongue and sponge simulators on the self-confidence building of midwifery students. Nurse Educ Pract.

[12] Sparks RA, Beesley AD, Jones AD (2006). “The sponge perineum”: an innovative method of teaching fourth-degree obstetric perineal laceration repair to family medicine residents. Fam Med.

[13] Wahyuningtyas R, Kurniawati EM, Utomo B (2022). Obstetrics and gynecology residents’ satisfaction and self-confidence after an anal sphincter injury simulation-based workshop in Indonesia: a pre- and post-intervention comparison study. J Educ Eval Health Prof.

[14] Shah R, Davidson A, Arnason M (2019). A novel approach to simulation-based perineal repair in undergraduate medical education. J Obstet Gynaecol Can.

[15] Banks E, Pardanani S, King M (2006). A surgical skills laboratory improves residents’ knowledge and performance of episiotomy repair. Am J Obstet Gynecol.

[16] Siddiqui NY, Stepp KJ, Lasch SJ (2008). Objective structured assessment of technical skills for repair of fourth-degree perineal lacerations. Am J Obstet Gynecol.

